# Mitomycin C and Vinorelbine for second-line chemotherapy in NSCLC – a phase II trial

**DOI:** 10.1038/sj.bjc.6603683

**Published:** 2007-03-13

**Authors:** A Babiak, J Hetzel, F Godde, H-H König, M Pietsch, M Hetzel

**Affiliations:** 1 Department of Internal Medicine II, Division of Pulmonary Medicine, University Medical Center, Ulm, Germany; 2 Health Economics Research Unit, University of Leipzig, Leipzig, Germany; 3 Medac, Wedel, Germany

**Keywords:** lung cancer, NSCLC, chemotherapy, second-line therapy, mitomycin, Vinorelbine

## Abstract

Single-agent therapy with Docetaxel or Pemetrexed is the current therapy of choice for second-line treatment in advanced non-small-cell lung cancer (NSCLC). The role of older agents was underattended over the last years. This study presents the combination of Mitomycin C and Vinorelbine in pretreated patients. Forty-two patients (stage IIIB and IV, pretreated with platinum-based chemotherapy) received 8 mg m^−2^ Mitomycin C on day 1 and 25 mg m^−2^ Vinorelbine on days 1 and 8 of a 28-day cycle. End points were objective tumour response, survival, and toxicity. Additionally, quality of life (QoL) was assessed. Five patients (11.9 %) achieved partial responses and 13 patients (31.9%) stable disease. Progression-free survival was 16 weeks. The median overall survival was 8.5 month. Eleven patients (26.2 %) suffered from grade 3 or 4 neutropenia and four patients (9.52%) from grade 3 or 4 anaemia. Evaluation of QoL showed that some items ameliorated during therapy. The therapeutic concept including Mitomycin C and Vinorelbine offers an efficacious and well-tolerated regimen, with relatively low toxicity. Objective response and survival data correlate with other second-line studies using different medication. As costs of Mitomycin C and Vinorelbine are lower compared with current drugs of choice, this regimen is likely to be cost-saving.

Lung cancer is the most common cancer in the world today (12.3% of all new cases), with an estimated 1.2 million new cases and 1.1 million deaths (17.8% of all cancer deaths) worldwide in 2000. Non-small-cell lung cancer accounts for approximately 80% of all cases of lung cancer (Landis *et al*, 1998). For chemotherapy-naive patients with a good performance status (PS) and stage IIIb or IV disease, platinum-based chemotherapy offers a modest survival advantage over best supportive care (BSC) alone (Grilli *et al*, 1993; Non-small Cell Lung Cancer Collaborative Group, 1995). Docetaxel was the first of the US Food and Drug Administration and European Agency for the Evaluation of Medical Products-approved chemotherapy agent for the second-line treatment of advanced NSCLC. The approval was based on two phase III studies (Fossella *et al*, 2000a; Shepherd *et al*, 2000). For patients with a good PS at the time of disease progression following first-line chemotherapy, docetaxel, despite a low response rate, is associated with a 10–20% increase of 1-year survival and an improved quality of life when compared with ifosfamide, Vinorelbine, or BSC alone. In 2004 and 2005, the two new substances Pemetrexed and Erlotinib received Food and Drug Administration approval for the second-line treatment of locally advanced or metastatic NSCLC. The median survival time ranges between 6.7 and 8.3 months. In view of the modest results of these drugs, other agents with single-agent activity in NSCLC are greatly needed for this patient population. An additional important factor is cost effectiveness that gains increased attention among healthcare systems in many countries. This phase II trial focused on the efficacy and tolerability of Mitomycin C in combination with Vinorelbine in pretreated patients suffering from NSCLC. The trial shows the application of a regimen, which is established and frequently used for patients with advanced breast cancer.

## MATERIALS AND METHODS

### Patient eligibility

This trial was conducted at our institution between January 2002 and April 2005. Eligible patients had locally advanced or metastatic NSCLC that had progressed during or after prior chemotherapy regimen. Before study entry, a minimum of 21 days must have elapsed since any prior chemotherapy. Patients may have had either measurable or assessable lesions. Eastern Cooperative Oncology Group performance status of 0–2 was required, as was adequate bone marrow (absolute granulocyte count of ⩾1.5 × 10^9^ cells l^−1^ and platelet count of ⩾100 × 10^9^ cells l^−1^), hepatic (total bilirubin level within normal limits, alkaline phosphatase level ⩽5 times the upper limit of normal, and serum transaminase ⩽1.5 times the upper limit of normal), and renal (serum creatinine level ⩽2.0 mg dl^−1^ or creatinine clearance ⩽60 ml min^−1^) function. No restriction was placed on the number of prior chemotherapy regimens or the amount of prior chemotherapy. Patients with prior Mitomycin C or Vinorelbine treatment were not included. Patients who had received prior radiation therapy were eligible provided that at least 30 days had elapsed from the completion of radiation to study entry. Patients with treated brain metastases were eligible provided that they were neurologically stable. All patients provided written informed consent. The study was approved by local institutional review boards and was conducted in compliance with institutional review board regulations.

### Treatment plan

Eligible patients were assigned to receive the combination of Mitomycin C and Vinorelbine. Patients were stratified according to stage of disease (stage IIIB or IV) and performance status (0 or 1 *vs* 2).

Patients received 8 mg m^−2^ Mitomycin C as a 10-min intravenous infusion on day 1 and 25 mg m^−2^ Vinorelbine as a 10-min intravenous infusion on days 1 and 8 of a 28-day cycle. Chemotherapy was given over six cycles or until disease progression, unacceptable toxicity, or until the patient or the investigator requested therapy discontinuation. Patients were instructed to take dexamethasone (4 mg orally twice daily the day of, and the day after each chemotherapy infusion). Ondanstrone was administered on the day of and the day after chemotherapy infusion.

Haematopoietic growth factors were not used prophylactically but were permitted therapeutically at the discretion of the treating physician. Prophylactic antiemetics were permitted.

The baseline assessment included a history and physical examination, complete blood count, comprehensive blood chemistries, calculated creatinine clearance, and computed tomography scan of the chest. Bone scans and brain imaging were performed only if clinically indicated.

The Lung Cancer Symptom Scale (LCSS) was administered at baseline and weekly during the study. The observer LCSS was administered at baseline and at the end of each cycle. Toxicity evaluations were based on the National Cancer Institute CTC, version 2. Haematologic laboratory values were evaluated weekly. Chemistry laboratory values were evaluated following days 1 and 8 of each cycle. Tumour measurements were assessed after every two cycles.

### Evaluation of response

Tumour responses were assessed radiographically every two cycles. Designations of complete response, partial response, no change, and progressive disease were based on the standardised response definitions established by the World Health Organization. Duration of response and time to progression were calculated as time from enrollement to the first objective evidence of tumour progression. Survival was calculated from the date of recruiting until death. Patients were treated for a minimum of two cycles (unless this was precluded by unacceptable toxicity or rapid disease progression). Patients with response or stable disease continued treatment for at least six cycles unless there was disease progression or unacceptable side effects. Patients who were responding or had stable disease could receive more than six cycles if they were achieving continued clinical benefit as determined by the treating physician. Patients with disease progression, unacceptable toxicity, treatment delay of more than 3 weeks, or intercurrent conditions that precluded continued treatment were removed from the study. On removal from study, patients were to be observed every 2 months until death to assess adverse events, quality of life, disease status, and survival.

### QOL assessment

QoL assessment was scheduled to be carried out at baseline and after drug treatment end. The validated instrument was the EORTC QLQ-C30 instrument with the LC 13 lung cancer module.

The instrument consists of a core questionnaire incorporating a global health and QoL scale, five multiitem function scales (physical, role, cognitive, emotional, and social), three multiitem symptom scales (fatigue, pain, nausea, and vomiting), and six single-item symptom measures (dyspnea, insomnia, appetite loss, constipation, diarrhea, and financial difficulties).

For each item, a linear transformation is applied to standardise the raw score to a range from 0 to 100, with 100 representing the best possible function/QoL, and highest burden of symptoms. All 30 items are rated by the patient.

### Statistical analysis

The primary objective of the study was to evaluate response rate, median survival, and time to progression. Secondary objectives were toxicities (including use of concomitant supportive measures), time to progressive disease (TPD), time to treatment failure (TTF), time to response, duration of response, and quality-of-life measurements (using the LCSS).

The number of patients needed in this trial was determined according to the optimal two-stage design for phase II studies proposed by Simon *et al*. (1989).

The null hypothesis that the remission rate is 5% was tested against the alternative hypothesis that the remission rate is at least 20%. If the null hypothesis is true, then the probability of erroneously concluding that the therapy is sufficiently promising (type I error) was limited to 5%. If the alternative hypothesis is true, the probability of erroneously rejecting the therapy for further study (type II error) should be less than 20%.

With these constraints, the maximum required sample size was 37 evaluable patients. The first stage consisted of 17 patients. If the number of responses after completing the first stage is 0, the trial could be terminated owing to futility.

Statistical analyses were performed by the independent institute GEM (Gesellschaft für Evaluation und Qualitätssicherung in der Medizin, Meerbusch, Germany) using the statistical packages SAS (SAS Institute, Cary, NC, USA), version 8.

## RESULTS

### Patient characteristics

A total of 42 patients were enrolled into the study. Median age was 63.9 (50–76) years. Seventeen per cent of the recruited patients had stage IIIB disease and 83% of the patients had stage IV disease. Demography is given in Table 1. All enrolled patients were assessable for objective response and survival analyses. All patients received treatment after inclusion into the study. Patients had performance status of 0 or 1 despite their extensive prior therapy.

### Treatment administration

Eighteen patients (43 %) received six cycles of chemotherapy and completed the whole course. Two patients (4.7%) and 16 patients (38.1%) were withdrawn from the study after one, respectively, and two cycles of chemotherapy owing to toxicity or progression. At a patient's request, therapy with Mitomycin C and Vinorelbine could be continued for further cycles out of the study.

### Efficacy

Objective response was assessed in 42 patients with histologically confirmed NSCLC, who received at least two chemotherapy infusions after inclusion. Partial response was observed in five patients (11.9%). In those patients, PR was observed after two cycles. After completion of six cycles, 13 patients (31.9%) were still in stable disease. Progression-free survival was 16 weeks (CI 95% 2.6–31.1 weeks). Median survival was 8.5 month (CI 95% 4.2–12.8 months).

### Safety and toxicity

Toxicity could be assessed in all 42 patients. Two patients were reported to have neither haematological nor non-haematological toxicity. Eleven patients (26.2%) had grade 4 neutropenia and four patients (9.5%) suffered from grade 3 or 4 anaemia. Grade 3 or 4 nausea or vomiting occurred in four patients (9.5%). Five patients (11.9%) received RBC transfusions (⩽2) among them only one (2.4%) required transfusion because of therapy. One patient obtained platelet transfusion. Filgrastim had not to be administered.

### Quality of life

A total of 30 patients (71.4%) completed the QLQ-C 30 questionnaire at the beginning of treatment, 25 patients (59.5%) answered the questionnaire additionally at drug treatment end. For patients who missed to complete the final questionnaire, the score was evaluated as last score carried forward. Global healthcare decreased from baseline 49.4 to last assessment value 40.8 (−17.4%). The mean score for nausea, insomnia, and constipation ameliorated during therapy.

## DISCUSSION

Survival benefits in patients with advanced NSCLC with the administration of platinum-based chemotherapy are modest in general compared with BSC (Fossella, 2000a, 2000b). Virtually all patients with advanced disease, however, ultimately develop disease progression after first-line therapy, and many such patients who maintain a good performance status are offered the option of second-line treatment (Lara *et al*, 2002). Many trials of second-line chemotherapy for NSCLC have been conducted to assess the efficacy of such therapy in second-line treatment. The most promising data were shown for chemotherapy with docetaxel and Pemetrexed (Hanna *et al*, 2004). Those drugs showed consistently good survival data in pretreated patients and represent the treatment of choice in second-line setting (Fossella *et al*, 1995, 2000b). Over the last years, also newly developed drugs such as EGFR or VEGFR blocking agents were introduced for second-line treatment.

The combination of Mitomycin C and Vinorelbine was studied extensively in patients suffering from breast cancer (Agostara *et al*, 1994; Vici *et al*, 1996). Several studies for second-line treatment were conducted in the past. Most studies used doses and intervals as we used here. Doses of up to 10 mg m^−2^ Mitomycin C were tested there. The idea of implementing Mitomycin C in the treatment of NSCLC has been generated as early as 1985 (Shinkai *et al*, 1985; Beck *et al*, 1987; Botto *et al*, 1989). In those times, treatment of NSCLC using chemotherapy was new and still diversely discussed (Non-small Cell Lung Cancer Collaborative Group, 1995). By then Mitomycin C was combined with either platinum-containing drugs or vinca-alcaloids. Several small studies elucidated the doublet combination of Mitomycin C and vinca-alcaloids as shown in Table 2.

Comparing earlier data and data from the present study allow several careful statements. All trials – including the present as well – investigated relatively small groups of patients. Also the treatment regimens varied slightly from the form applied today only one trial focused on second-line treatment (Kris *et al*, 1985). Data that could be generated with those former trials correlate with today's rates in many ways.

In terms of response rates and survival data – which must be handled carefully because of small trial size – all trials showed data, that compete with today's second-line regimens. Toxicity is limited to haematologic toxicity in several cases; however, the chemotherapy is tolerated well, showing a low incidence of patients disabling toxicities such as nausea, emesis, alopecia and fatigue. Those data can also be compared with data from breast cancer studies, which show similar rates of toxicity.

Tolerability of this regimen should be focused also in terms of dose intensity. Milleron *et al* (1991) could show a median number of 10 chemotherapy cycles given per patient. This could be proven in the present trial, in which over 40% of the patients completed all six planned cycles of treatment. Toxicity was also comparable to today's data with grade 3/4 anaemia of 8.3% and leucopenia of 33.3%.

Treatment over six cycles was shown to be possible and was performed in some patients out of the study protocol. A known side-effect of Mitomycin C is the haemolytic uremic syndrome (HUS). One patient suffered from HUS after given 10 cycles of the combination, but recovered completely. Haemolytic uremic syndrome is thought to occur at a cumulative dose of ⩾60 mg m^−2^ of Mitomycin C; however, the relation between dose and occurrence could not be shown persistently in the literature. Early detection of HUS by screening for hemolysis and schistocytosis might be helpful.

QoL assessment was also implemented into this study. Without control group conclusions could only drawn from a comparison to historic data focusing also onto second-line treatment. An earlier trial comparing Docetaxel *vs* BSC showed that treatment gave a moderate decrease for several items (pain: −12%, physical function: −19%, global health status: −21%) (Dancey *et al*, 2004). In our study, the decrease was comparable with the items physical function: −9.3% and global health status: −17.4%) The item pain was difficult to compare because of different baseline scores. Thus Dancey reported a decrease from baseline score 80–68, whereas in our study the very low baseline of 30 moderately increased to 36.7. The difference is possibly due to different strategies of pain management that lead to incomparable values.

Compared with already published data, our results also underline the good tolerability of the combination therapy. Taking those data into account, this regimen fulfils requirements of a modern second-line treatment in terms of objective tumour response, survival and quality of life benefit.

Being one of the most frequently diagnosed cancers, NSCLC causes an enormous economic burden for healthcare systems worldwide. As in many patients NSCLC is only eligible for palliative chemotherapy treatment, drug costs are a major driver of treatment costs in Europe. Therefore, it should be pointed out that the costs of Mitomycin C and Vinorelbine are considerably lower than those of the more recently developed drugs of choice for second-line treatment. Based on German pharmacy prices (Rote Liste 2006), the costs per cycle of Mitomycin C (8 mg m^−2^) and Vinorelbine (2 × 25 mg m^−2^) are 574 EUR compared with 1802 EUR for Docetaxel (25 mg m^−2^) and 3641 EUR for Pemetrexed (500 mg m^−2^), assuming a body surface of 1.8 m^2^; oral Erlotinib (150 mg day^−1^) costs 2590 EUR per month. Thus, within six cycles, more than 18 000 EUR could be saved by using Mitomycin C/Vinorelbine instead of Pemetrexed. Although cost-effectiveness is not only the motivation to choose a specific kind of therapy, it will have to be considered increasingly when setting priorities in collectively financed health care systems.

In times of targeted therapy, mitomycin might become of particular interest in the future. It was shown recently that bclxl and Bcl-2 modulate the chemosensitivity against various drugs such as mitomycin. Influencing the activity of these genes, for example using antisense oligunucleotides, could already show experimentally to enhance the cytostatic effects of mitomycin (Emi *et al*, 2005). It has been also documented that the presence of Fanconi anemia/BRCA2 mutations in pancreatic cells is predictive for sensitivity to Mitomycin, which causes DNA–interstrand crosslinking (van der Heijden *et al*, 2005). Those mutations could also be found in NSCLC patients and might be influencing sensitivity to mitomycin therapy (Marsit *et al*, 2004).

Treatment using Mitomycin C and Vinorelbine for pretreated patients with metastatic or locally advanced NSCLC was introduced already almost 20 years ago, but was not used widely over the last years. Data show promising efficacy data, toxicity is modest and treatment costs are low. Nowadays it is difficult to develop new treatment strategies or to change existing ones. The currents guidelines are based on fairly large trials and show acceptable results. Using older regimens should be followed anyway. One cohort of patients which is still not treated with full satisfaction is the group of older patients or people with reduced PS or significant comorbidity. Those patients – and to a lesser extend all treated patients – should be allowed to receive well-tolerable cytostatic therapy. The recent introduction of Pemetrexed and the use of small molecules for second-line treatment are yielding for that direction. Most of the newly launched trials try to focus on QoL issues and raise the concept of symptom free plus progression free survival. Owing to its excellent tolerability, the combination of Mitomycin C and Vinorelbine might have its destination in patients with reduced PS of two or lower.

## Figures and Tables

**Table 1:** Patients characteristics

**Characteristic**	**(%)**
Sex	
Male	83.3
Female	16.67
Age (years)	
Median	64
Range	42–76
Performance status	
0 or 1	95
2	
Prior platinum	100
Prior taxane	14.3
Best response, any prior chemotherapy	
CR/PR	14.3
SD	33.3
PD/unknown	50/2.4
Time since last chemotherapy	
<3month	71.4
Histology	
Adenocarcinoma	50
Squamous cell carcinoma	40.47
Prior radiation	11.9

CR, complete remission; PD, progressive disease; PR, partial response; SD, stable disease.

**Table 2:** Mitomycin in combination with vinorelbine, vindesine, or vinblastine in NSCLC

**Author date**	**Chemotherapy line**	**Dose**	**Evaluable patients**	**OR (%)**	**Median survival (weeks)**
Our study	Second	Mito 8 mg m^−2^ d1 q4w Vino 25 mg m^−2^ d1, 8 q4w	42	11.9	37
Gralla *et al* (1994)	First	Mito 8 mg m^−2^ d1 q4w Vino esc. 25–35 mg m^−2^ (phase I)	42	34	43.5
Milleron *et al* (1991)	First	Mito 6 mg m^−2^ d1 q3w Vino 25 mg m^−2^ d1, 8, 15 q3w	21	23.8	—
Gatzemeier *et al* (1991a)	First	Mito 10 mg m^−2^ d1 q4w Vind 3 mg m^−2^ d1, 8 q4w	66	22.7	23
Gatzemeier *et al* (1991b)	First	Mito 10 mg m^−2^ d1 Vind 3 mg m^−2^ d1, d8 (max. 5 mg)	58	22.4	27.7
Luedke *et al* (1990)	First	Induction: Vind/Mito then Mito 15 mg m^−2^ d1 q6w Vind 3 mg m^−2^ d1, 15, 29 q6w	122	27	20.4
Shinkai *et al* (1985)	First	Induction: Vind/Mito then Mito 8 mg m^−2^ d1 q3w Vind 3 mg m^−2^ d1 q2w	30	10	44.3
Kris *et al* (1985)	First	Induction: Vind/Mito then Mito 10 mg m^−2^ d1 q6 to 8w Vind 3 mg m^−2^ q2w	55	36	26.5
Kris *et al* (1985)	Second	Induction: Vind/Mito then Mito 10 mg m^−2^ d1 q6 to 8w Vind 3 mg m^−2^ q2w	29	17	21.3
Ruckdeschel *et al* (1984)	First	Mito 10 mg m^−2^ d1 q3w Vinb 6 mg m^−2^ d1 q3w	101	13	18

OR, overall response; Mito, mitomycin; Vinb, vinblastine; Vind, vindesine; Vino, vinorelbine.
